# ‘*Involve me and I learn*’: an experiential teaching approach to improve dyspnea awareness in medical residents

**DOI:** 10.1080/10872981.2022.2133588

**Published:** 2022-10-11

**Authors:** Maxens Decavèle, Laure Serresse, Frédérick Gay, Nathalie Nion, Sophie Lavault, Yonathan Freund, Marie-Cécile Niérat, Olivier Steichen, Alexandre Demoule, Capucine Morélot-Panzini, Thomas Similowski

**Affiliations:** a Sorbonne Université, INSERM, UMRS1158 Neurophysiologie Respiratoire Expérimentale et Clinique, Paris, France; b AP-HP, Groupe Hospitalier Universitaire APHP-Sorbonne Université, site Pitié-Salpêtrière, Service de Médecine Intensive, Réanimation, Département R3S, Paris, France; c AP-HP, Groupe Hospitalier Universitaire APHP-Sorbonne Université, site Pitié-Salpêtrière, Unité Mobile de Soins Palliatifs, Paris, France; d AP-HP, Groupe Hospitalier Universitaire APHP-Sorbonne Université, site Pitié-Salpêtrière, Laboratoire de parasitologie-mycologie, Paris, France; e AP-HP, Groupe Hospitalier Universitaire APHP-Sorbonne Université, site Pitié-Salpêtrière, Département R3S, Paris, France; f AP-HP, Groupe Hospitalier Universitaire APHP-Sorbonne Université, site Pitié-Salpêtrière, Service d’accueil des urgences, Paris, France; g Sorbonne Université, INSERM, UMRS 1166, IHU ICAN, Paris, France; h AP-HP, Groupe Hospitalier Universitaire APHP-Sorbonne Université, site Tenon, Service de médecine interne, Paris, France; i Sorbonne Université, INSERM, UMRS 1142 LIMICS, Paris, France; j AP-HP, Groupe Hospitalier Universitaire APHP-Sorbonne Université, site Pitié-Salpêtrière, Service de Pneumologie, Département R3S, Paris, France

**Keywords:** Experiential learning, dyspnea, experimental dyspnea, personal experience, medical trainees, medical residents, symptoms experience, respiratory suffering, empathy enhancing

## Abstract

**Background:**

Dyspnea is a frightening and debilitating experience. It attracts less attention than pain (‘dyspnea invisibility’), possibly because of its non-universal nature. We tested the impact of self-induced experimental dyspnea on medical residents.

**Materials and Methods:**

During a teaching session following the principles of experiential learning, emergency medicine residents were taught about dyspnea theoretically, observed experimental dyspnea in their teacher, and personally experienced self-induced dyspnea. The corresponding psychophysiological reactions were described. Immediate and 1-year evaluations were conducted to assess course satisfaction (overall 0–20 grade) and the effect on the understanding of what dyspnea represents for patients.

**Results:**

Overall, 55 emergency medicine residents participated in the study (26 men, median age 26 years). They were moderately satisfied with previous dyspnea teaching (6 [5–7] on a 0–10 numerical rating scale [NRS]) and expressed a desire for an improvement in the teaching (8 [7–9]). Immediately after the course they reported improved understanding of patients’ experience (7 [6–8]), which persisted at 1 year (8 [7–9], 28 respondents). Overall course grade was 17/20 [15–18], and there were significant correlations with experimental dyspnea ratings (intensity: r = 0.318 [0.001–0.576], p = 0.043; unpleasantness: r = 0.492 [0.208–0.699], p = 0.001). In multivariate analysis, the only factor independently associated with the overall course grade was ‘experiential understanding’ (the experimental dyspnea-related improvement in the understanding of dyspneic patients’ experience). A separate similar experiment conducted in 50 respiratory medicine residents yielded identical results.

**Conclusions:**

This study suggests that, in advanced medical residents, the personal discovery of dyspnea can have a positive impact on the understanding of what dyspnea represents for patients. This could help fight dyspnea invisibility.

## Introduction

Dyspnea is a ubiquitous symptom of cardiac or respiratory dysfunction and of many other disorders. It stems from an upsetting or distressing awareness of breathing sensations [1] that has been defined as ‘*a subjective experience of breathing discomfort made of various sensations that can vary in intensity*’ [1]. Acute dyspnea is a frightening experience that is very common, being reported by more than 15% of patients at hospital admission [2], 50% of patients seen in the emergency room [2], and up to 50% of critically ill mechanically ventilated patients [3,4]. It independently predicts hospital death [5] and post-intensive care stress disorders [3]. Chronic dyspnea, in turn, shapes the lives of affected patients, for whom it represents a major physical, psychological, and social burden. Given the epidemiology of chronic respiratory diseases, congestive heart failure and neuromuscular disorders, chronic dyspnea affects millions worldwide.

Dyspnea, however, attracts much less attention from professional caregivers than, for example, pain [6,7]. Dyspneic patients report an insufficient understanding of their experience by others that aggravates their suffering [8]. For their part, caregivers find it difficult to talk with their patients about dyspnea [9]. This defines ‘dyspnea invisibility’ [10], which limits access to due care [11] and therefore raises human rights issues [12]. Among many factors, the lack of universality of dyspnea can contribute to dyspnea invisibility. ‘Pathological breathlessness’[13], i.e., the anxiogenic breathing difficulties due to a constraint that cannot be controlled, as opposed to the non-threatening ‘healthy breathlessness’ that occurs during sport, is a far less common experience than pain. The absence of a personal reference system can make it difficult to understand the suffering associated with dyspnea and could lead to empathic distress and avoidance behaviors [14]. Furthermore, it has been reported that caregivers who witness troubled breathing can themselves experience so-called ‘vicarious dyspnea’ and malaise [15,16]. In the case of medical or paramedical trainees, insufficient dyspnea-targeted teaching is another factor that can play a role in dyspnea invisibility.

We hypothesized that the implementation of a dyspnea course for medical residents, designed according to Kolb’s experiential learning cycle [17] and including personal exposure to experimental dyspnea, would make them associate the concept of dyspnea with precise sensory and emotional mental imagery (embodied cognition [18]) and, therefore, would change their perception of the lived experience of dyspneic patients. This hypothesis was based on anecdotal observations of the impact of laboratory dyspnea on medical personnel (after his first personal encounter with air hunger, a young intensivist from our group once said that he would ‘*never again tell an agitated [dyspneic] mechanically ventilated patient to calm down*’ [19]). A population survey also demonstrated that the breathing discomfort associated with COVID-19 face masks can enhance the general public understanding of the lived experience of chronic respiratory disease patients [20]. More generally, the exposure of medical trainees to symptoms leads to improved empathy toward patient experiences experiential learning, see [21–23].

**Figure 1.**
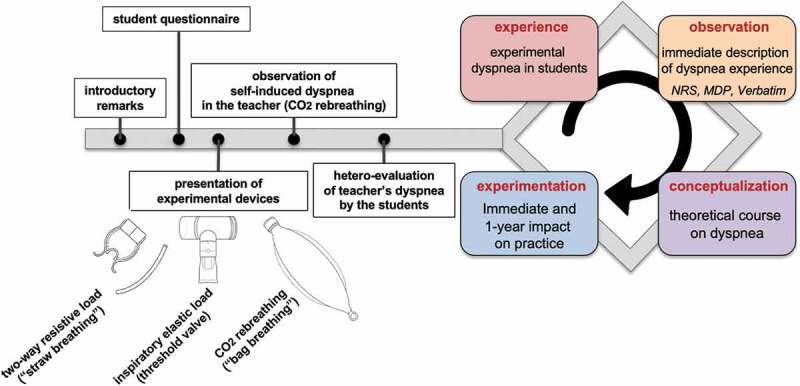
Course sequence. The design of the course was based on the four-stage process of Kolb’s experiential learning cycle [17], the students’ knowledge about dyspnea being enriched through personal experience of dyspnea. After being engaged in an actual dyspnea experience (step 1: concrete experience), students were invited to reflect on what happened to them during this experience (step 2: reflection/observation). A theoretical course then intended to help the students amalgamate their personal experience and theoretical concepts (step 3: conceptualization/explanation). Finally, immediate, and delayed evaluations assessed the change in the students’ beliefs and attitudes about dyspnea (step 4: active experimentation/projection).

To test this hypothesis, we designed a teaching session (Figure 1), during which residents were taught about dyspnea from a theoretical point of view (‘tell me’), confronted by the experimental dyspnea of a healthy subject (‘show me’), and were themselves exposed to short bouts of dyspnea induced by different means (‘involve me’).

## Materials and methods

### Design and setting

This pilot study was designed by an intensivist (MD), a palliative care specialist (LS), a psychologist (SL), and two respiratory physicians (CMP, TS), all of whom were engaged in academic teaching and involved in clinical and experimental dyspnea research, and three of whom (MD, LS and CMP) were qualified in medical pedagogy. The two main investigators (MD and LS) conducted the study at the Faculty of Medicine, Sorbonne Université, Paris, France, in the form of a 90 minute session with a follow-up at 1 year. The study was first conducted in a homogeneous group of residents in the emergency medicine specialization cursus who had completed 8 years of medical studies and were entering their ninth and final year. Corroboration of the results was then sought for in a second group of respiratory medicine residents. The first group was included in January 2020 and the second group was in April 2022, the 2-year period between groups being due to the COVID-19 pandemic.

The study was approved by the university ethical committee (*Comité d’Éthique de la Recherche*, Sorbonne Université, Paris, France, # CER 2020–2). Regarding information, the participants were provided with a detailed description of the study. Its background and design were first explained orally, after which the residents were given a printed information leaflet. They were clearly informed that the experiential part of the course involved enduring some degree of physical and psychological discomfort (e.g., transient headache or anxiety), that no residual untoward effects were to be expected, and that they would have full control of their dyspnea through the possibility of stopping the experiment at any moment without any justification. They were also informed that, in the event of abnormally strong or residual psychological reactions, they could contact the investigators or a psychologist (contact information provided in the leaflet), for access to support at any time. Regarding consent, the participants were told orally and in writing that they were free not to participate in any part of the course (with no questions asked) and, in this case, to either observe or leave (without any consequence on their marks or graduation). Taking part to the course in general and its experiential part in particular was therefore considered as explicit consent.

## Teaching sequence

The course was organized as follows (Figure 1):

1) Introductory remarks, including the terms of the upcoming course (5 minutes);

2) Completion of a generic questionnaire exploring individual characteristics, academic training, personal history, attitudes, and beliefs regarding dyspnea and pain, and satisfaction with previous dyspnea teaching; (5 minutes; Table 1 and electronic supplement ES1); the participants were also asked to evaluate their self-perceived degree of empathy on a 10 cm visual analogue scale (from ‘not empathetic at all’ to ‘extremely empathetic’); participants who rated their self-perceived level of empathy 7 or more were allocated to a ‘high empathy’ group, those who gave a rating of 6 or less were allocated to a ‘low empathy’ group;

**Table 1.** Data collected at the different time points of the dyspnea course.

Before the beginning of the dyspnea course	
***Generic student questionnaire (electronic supplement ES1)***	
Age, gender, personal medical history	
Personal experience of pain	• yes/no; if yes 0–10 NRS
Personal experience of ‘pathological breathlessness’	• yes/no; if yes 0–10 NRS
Self-perceived degree of empathy	• 0–10 NRS ^(1)^
Dyspnea teaching received during medical studies	• number of courses • 0–10 NRS rating of overall satisfaction • 0–10 NRS rating of desire for more teaching
Self-perceived confidence in managing pain upon emergency room admission	• 0–10 NRS
Self-perceived confidence in managing dyspnea upon emergency room admission	• 0–10 NRS
Attitudes and habits regarding the management of dyspnea and pain in clinical practice	See details in ES1
**Psychophysiological reactions to the teacher’s dyspnea***(electronic supplement ES2)*	
Intensity of the teacher’s dyspnea?	• 0–10 NRS
Did you experience dyspnea yourself while watching the teacher’s dyspnea?	• yes/no • if yes, 0–10 NRS
**Psychophysiological reactions to experimental dyspnea***(electronic supplement ES3)*	
Intensity of your dyspnea ?	• 0–10 NRS
Multidimensional Dyspnea Profile ^(2)^ (French version) ^35^	• see details in ES3
Verbatim (write two sentences summarizing your experience)	• lexicometric analysis
**At the end of the teaching session***(electronic supplement ES4)*	
To what extent did your personal experience of dyspnea make you better understand what dyspneic patients feel?	• 0–10 NRS
Grade the course in general	• 0–20 mark
**One year after the teaching session***(electronic supplement ES5)*	
Online 4 questions survey	• see details in ES5
Three-word verbatim to describe the impact of the course	

NRS, numerical rating scale

*^(1^) participants who rated their self-perceived level of empathy 7 or more were allocated to a ‘high empathy’ group, those who gave a rating of 6 or less to a ‘low empathy’ group.*

^(2)^*a multidimensional instrument measuring the intensity of dyspnea unpleasantness (A1) and the sensory (SQ) and affective (A2) dimensions of dyspnea; (electronic supplement, ES3).*

3) Presentation of the experimental devices (3 minutes; see below);

4) Demonstration of self-induction of experimental dyspnea by the teacher using a CO_2_ rebreathing bag (2 minutes); the end-tidal partial pressure of CO_2_ was measured continuously using an infrared capnometer (MicroStream®, Medtronic, Dublin, Ireland) with the display permanently visible to the participants;

5) Psychophysiological reaction to the teacher’s dyspnea (1 minute, Table 1 and electronic supplement ES2);

6) Self-induction of experimental dyspnea by the participants (2 minutes; a large screen stopwatch visible to the participants was placed on the teacher’s desk); the participants were randomly assigned to one of three modalities to self-induce dyspnea (we only had a stock of ten ITL):

*a) CO_2_ rebreathing*: the participants were asked to breathe in a 2 L rebreathing bag (single-use reservoir bag, Intersurgical, Wokingham, Berkshire, UK) directly connected to the mouth, with a nose clip on;

*b) Inspiratory threshold loading*: the participants were connected to a spring-loaded threshold valve (50 cmH_2_0 load) fitted with a 2-way valve to leave expiration free, with a nose clip on;

*c) Straw breathing*: the participants, wearing a nose clip, were asked to breathe using plastic tubing (8 cm extension tubing with a 3 mm inner diameter, Asept Inmed, Quint-Fonsegrives, France) directly connected to their mouth and providing both inspiratory and expiratory resistance.

7) Psychophysiological reactions to experimental dyspnea (Table 1 and electronic supplement ES3) (5 minutes, see details below);

8) Theoretical course on the pathophysiology of dyspnea, its clinical impact and its treatment (40 minutes);

9) Immediate completion of a satisfaction questionnaire evaluating the course (5 minutes, Table 1 and electronic supplement ES4), including an overall rating (from 0 to 20).

10) 1-year evaluation (Table 1 and electronic supplement ES5) (available only in the first group).

The design of the course respected the four-stage process of Kolb’s experiential learning cycle, the students’ knowledge about dyspnea being enriched through the transformation of an experience of dyspnea (Figure 1). After being engaged in an actual dyspnea experience (step 1: concrete experience), students were invited to reflect on what happened to them during this experience (step 2: reflection/observation). A theoretical course then intended to help the students amalgamate their personal experience and theoretical concepts (step 3: conceptualization/explanation). Finally, immediate and delayed evaluations assessed the change in the students’ beliefs and attitudes about dyspnea (step 4. Active experimentation/projection) [17].

## Collected variables and empathy assessment

Table 1 summarizes the data collected at the different time points.

## Statistical analysis

The data analysis followed a 3-part statistical plan: 1) the psychophysiological reactions to the observation of the teacher’s demonstration of dyspnea and to the personal experience of dyspnea were described and compared between the ‘low empathy’ and ‘high empathy’ participants, 2) the psychophysiological reactions to the personal experience of dyspnea were described and compared between modalities of self-induced dyspnea, 3) factors associated with the overall grade given to the course were identified.

For these analyses, continuous variables are described with median and interquartile range and compared with nonparametric tests (Mann-Whitney for comparisons between two groups [e.g., dyspnea ratings between high- and low-empathy participants], Kruskall-Wallis for comparisons between more than two groups [e.g., dyspnea ratings across experimental devices]). Categorical variables are described as percentages and compared using the Chi [2]-test or Fisher’s exact test as appropriate. Correlations (Spearman) were calculated between the overall grade given to the course by the participants and variables considered likely to be related to this grade. The subset of these variables with a corrected p-value below 0.2 was retained for inclusion in multivariate analysis, and tested with three different approaches, namely multiple linear regression, stepwise multivariate regression and partial least square (PLS) regression. Based on the root mean square error (RMSE) and on the adjusted R^2^, multiple linear regression was retained as the best approach. P-values below 0.05 were considered statistically significant.

A lexicometric analysis was performed on the verbatim of the participants immediately after their exposure to dyspnea. Details are reported in the electronic supplement (ES6). A word cloud was generated to represent the 3-word verbatim collected at 1-year (for the first group only).

Analyses were performed using R version 3.6.1.

## Results

### Participants

Fifty-five emergency medicine residents, representing the first group, participated in the study (median age 26, interquartile range [26–27] years; 26 men [47%]) (Table 2).

**Table 2.** Participant characteristics.

Variables	Whole n = 55	Low-empathyn = 13	High-empathyn = 42	*P*
General characteristics				
Age, years	26 (26–27)	26 (26–28)	26 (25–27)	0.113
Gender (male), *n (%)*	26 (47)	9 (69)	17 (40)	0.069
Level of self-reported empathy, *NRS*	7 (7–8)	4 (4–5)	7 (7–8)	**<0.001**
Personal experience of pain*, *n (%)*	47 (85)	10 (77)	37 (88)	0.318
Rating of past experience of pain, *NRS*	8 (7–8)	8 (7–8)	7 (7–8)	0.713
Personal history of dyspnea* (pathological breathlessness), *n (%)*• asthma attacks, *n (%)*• fear of drowning, *n (%)*• miscellaneous, *n (%)*	17 (31) 11 (20) 3 (6) 3 (27)	2 (15) 2 (15) 0 (0) 1 (8)	15 (36) 9 (21) 3 (7) 2 (5)	0.303 1.000 1.000 0.562
Personal history of healthy breathlessness (sport), *n (%)*	4 (7)	2 (15)	2 (5)	0.234
Rating of past experience of pathological breathlessness, NRS	6 (5–6)	6 (6–8)	6 (4–6)	0.232

Continuous variables are expressed as median (interquartile range) and categorical variables are expressed as number (%).

NRS, numerical rating scale (from 0 minimal value to 10 maximal value)

* Participants who answered ‘yes’ to the ‘did you previously experience pain or dyspnea’, and provided both a description of the associated circumstance and a NRS rating.

‘Pathological breathlessness’[14] is defined as an experience of anxiogenic breathing difficulties due to a constraint that cannot be controlled, and correspond to the medical term ‘dyspnea’. ‘Healthy breathlessness’ is the non-threatening sensation that occurs in response to intense activities in normal individuals (e.g., sports, or sexual intercourse).

#### Personal history and characteristics

Table 2 provides the participant characteristics overall and for the ‘low empathy’ and ‘high empathy’ groups. Eighty-five percent (n = 47) answered ‘yes’ to the question regarding personal experience of pain, provided a description (mostly bone fractures, n = 17, and menstrual pain, n = 5), and provided the corresponding NRS rating (8 [7–8]; scale 0 [minimal]-10 [maximal]). Regarding previous experience of dyspnea (‘pathological breathlessness’), 17 participants (31%) answered ‘yes’, provided a description (asthma attacks, n = 11; fear of drowning at the swimming pool or in the sea, n = 3; miscellaneous, n = 3 [exercise during pregnancy, acute exposure to smoke, pneumonia]), and provided an NRS rating (6 [5–6]). Of note, 4 participants reported episodes of ‘healthy breathlessness’ during sport, which was not considered as dyspnea. Past experience of pain was significantly more frequently reported than past experience of dyspnea (p < 0.001), and the intensity of recalled pain was significantly greater than the intensity of recalled dyspnea (p < 0.001). Overall, the participants self-rated their empathy level 7 [7–8], with a significant difference between men and women (7 [6–8] vs. 8 [7–8], respectively, p = 0.032).

#### Knowledge about dyspnea

Among the 55 participants, 18% had no recollection of participating in any course regarding dyspnea, 21% recalled one course and 21% recalled two courses. Forty percent reported 3 or more courses. Those who reported one or more courses rated the quality of the dyspnea-related academic teaching received at 6 [5–7], and their wish to see improvement in this teaching at 8 [7–9].

#### Attitudes and beliefs towards dyspnea

Table 3 summarizes attitudes and beliefs toward pain and dyspnea management in patients on emergency room admission. The participants rated their self-confidence in dealing with dyspneic and painful patients at 6 [5–7] and 7 [5–8], respectively, (p = 0.445). As depicted in Figure S1 (electronic supplement ES7), the two main reasons for not using morphine significantly differed for the relief of pain (fear of negative effects on ventilatory control and fear of delirium) or dyspnea (priority given to other treatments aimed at correcting the causative phenomena, and the belief that the use of morphine for dyspnea is restricted to end-of-life care).

**Table 3.** Attitudes and beliefs toward pain and dyspnea management in patients on emergency room admission.

Variables	Pain	Dyspnea	*P*
Confidence with the management of the considered symptom, *NRS*	7 [5–8]	6 [5–7]	0.445
Propensity to systematically look for the symptom in patients on emergency room admission, *NRS*	9 [8–10]	8 [7–9]	**0.016**
How often do you prescribe morphine for symptom relief? Never Very rarely (1/year) Sometimes (1/month) More than sometimes (>1/month)	0 (0) 1 (2) 9 (16) 45 (82)	10 (18) 24 (44) 16 (29) 5 (9)	**<0.001**
The right to symptom relief is part of the public health code*, *n (%)*	55 (100)	29 (53)	**<0.001**
Notion that a clinically important threshold exists for the considered symptom, *n (%)*	48 (87)	11 (20)	**<0.001**
What is the proposed clinically important threshold for the considered symptom **^$^**	5 (3–6)	4 (3–5)	0.090
A symptom intensity rated ≥8 on NRS required immediate symptomatic relief, *n (%)*	52 (100)	50 (96)	0.879
Propensity to use morphine if symptom intensity ≥8, *NRS*	8 [7–9]	4 [2–6]	**<0.001**

Continuous variables are expressed as median (interquartile range) and categorical variables are expressed as number (%).

NRS, numerical rating scale (from 0 minimal value to 10 maximal value)

* The 2016 version of the French public health code (article L1110-5) states that everyone has the right to the relief of his/her ‘suffering’. This term replaces the word ‘pain’ that appeared in the 2002 version.

**^$^** The cut-off for clinically important pain that required prompt initiation of analgesic is 4 on a numerical rating scale ^36^

**^$^** There are no guidelines on the clinically important threshold of dyspnea. A cutoff of 4 has been proposed ^37^, but there is evidence that at least one third of patient rating their dyspnea ‘3’ consider this clinically intolerable ^38^.

## Psychophysiological reactions to the teacher’s dyspnea

Observing dyspnea elicited dyspnea (vicarious dyspnea) in 45 of the 55 participants (82%), rated 4 [2–5]. The participants rated the dyspnea of their teacher 6 [5–7], for a self-rating of 6 by the teacher. Participants in the ‘high empathy’ group reported vicarious dyspnea more frequently (88% vs. 62%, p = 0.030) and more intensely (4 [3–5] vs. 2 [0–5], p = 0.049) than those in the ‘low empathy’ group. Those in the ‘high empathy’ group also provided significantly higher estimates of the teacher’s dyspnea intensity (6 [5–7] *vs*. 5 [4–6], p = 0.046). Gender and personal history had no significant impact on observed dyspnea outcomes.

## Psychophysiological reactions to experimental dyspnea

Overall, 2/55 participants (4%) decided not to participate in the ‘experimental dyspnea’ phase of the course. Among the 53 remaining participants, 31 (59%) were randomized to straw breathing, 16 (30%) to CO_2_ rebreathing (30%), and 6 (11%) to inspiratory threshold loading. Fifty-two (98%) of those reported dyspnea in response to the stimulus, and 1 participant did not report dyspnea (straw breathing). Overall, participants were exposed to the stimulus for 60s [40–120 s] without any between-methods differences. The participants rated breathing discomfort intensity 6 [5–8] and breathing discomfort unpleasantness (A1 scale of the MDP) 8 [6–9]) (p < 0.001). A1 ratings were inversely correlated with the duration of exposure to the dyspnogenic stimulus: the more intense the dyspnea, the shorter the exposure (r = −0.533, 95% confidence interval (CI) [−0.701–-0.294], p < 0.001). ‘My breathing requires muscle work or effort’ and ‘I am breathing a lot’ were the two sensory descriptors the most frequently reported whereas “I am not getting enough air or I am suffocating or ‘I feel hunger for air’ was the most intensely reported. Anxiety and frustration were the two most frequently and intensely reported emotional descriptors. Overall MDP results are depicted in Figure S2 (ES8). Table S1 (ES9) and Figure S3 (ES10) provide MDP ratings according to the stimulus used. MDP ratings did not differ significantly between participants from the high or low empathy groups or according to gender.

## Evaluation of the course

### Immediate evaluation

The participants rated the experimental dyspnea-related improvement in the understanding of dyspneic patients’ experience (‘experiential understanding’ variable) at 7 [6–8]. They rated the course at 17 [15–18] out of 20, with a significant positive correlation between these two ratings (r = 0.704 95%CI [0.598–0.875], p < 0.001). Statistically significant correlations were also shown between the overall course rating and the intensity of breathing discomfort in response to self-induced dyspnea (r = 0.318 95%CI [0.001–0.576], p = 0.043), dyspnea unpleasantness (MDP A1, r = 0.492 95%CI [0.208–0.699], p = 0.001); MDP A2 (r = 0.324 95%CI [0.198–0.612], p = 0.039); MDP affective domain (r = 0.363 95%CI [0.212–0.636], p = 0.020); and MDP immediate perception domain (r = 0.342 95%CI [0.245–0.634], p = 0.029). Among these variables, only ‘dyspnea experience’ was retained as independently correlated with the overall course rating in the multivariate analysis.

### One-year evaluation

Fifty-three percent (28/53) of the participants having participated in the dyspnea course answered the one-year follow-up questionnaire. All of them considered that the course had been useful to better apprehend what dyspneic patients experience. They rated ‘*do you regard yourself more attuned than before to what acute dyspnea represents for the patients*’ at 8 [7–9], *‘do you feel more confident than before when managing acute dyspnea in the ER context?*’ at 7 [6–8], and ‘*do you experience less anxiety than before when managing a dyspneic patient in the ER context?*’ at 7 [5–8].

## Corroboration group

Fifty participants were included in the corroboration group (median age 27, interquartile range [26–28] years, 42% men). The prevalence of personal history of pain, dyspnea, or healthy breathlessness was 100%, 34%, and 12%, respectively. The participants self-rated their empathy level at 7 [7–8], and 28 (56%) were classified high-empathy. Vicarious dyspnea was reported by 38 participants (76%) and rated at 4 [2–5]; this was rated 5 [4–6] in the high empathy group and 3 [2–5] in the low empathy group (p = 0.054). Twenty-one participants (42%) were randomized to straw breathing, 24 (48%) to CO_2_ rebreathing (30%), and 5 (10%) to inspiratory threshold loading. MDP A1 ratings were inversely correlated with the duration of exposure to the dyspnogenic stimulus (r = −0.560 [−0.732–-0.320], p = <0.001). The ‘experiential understanding’ of dyspnea was rated at 7 [6–8] with an overall course grade of 18 [17–19], and there was a significant positive correlation between these two ratings (r = 0.512 [0.262–0.698] p < 0.001). There were also statistically significant correlations between the overall course rating and the intensity self-induced dyspnea (r = 0.456 [0.189–0.660], p = 0.001) and MDP A1 (r = 0.433 [0.161–0.644], p = 0.002). One-year assessment was not available for this group.

## Discussion

Before taking the dyspnea course described in this study, the participating emergency medicine residents considered that their prior training about dyspnea could be improved and expressed a desire for more advanced training. Immediately after the course and 1 year later, the participants reported an improved understanding of the experiences of dyspneic patients. The level of overall satisfaction with the course was high. Several arguments suggest that the personal experience of induced dyspnea was an essential driver of the positive reaction to the course.

### Use of the personal experience of dyspnea as a teaching tool

We incorporated experimental dyspnea in our teaching approach as an extension of Kolb’s experiential learning cycle that places personal implication at the heart of the learning process with the aim of connecting theory to reality [17,24] (Figure 1). The advent of simulation in medical education and its success with medical trainees [25,26] has heavily relied upon this type of approach. Similarly, educational interventions requiring healthcare providers to ‘become’ a patient (namely to live the patients’ symptoms, like visual impairment or auditory hallucinations [21–23]) appear particularly successful at increasing the understanding and recognition of the patient’s experience [27]. Our study seems to be the first to test this type of approach in the respiratory domain. Indeed, existing dyspnea-oriented teaching instruments and courses (like the educational dimension of the ‘*Breathing, Thinking, Functioning*’ clinical model [28,29]; see also [30,31]) can change participants’ previous beliefs and attitudes, but they do not involve experiential aspects. One could argue that inducing respiratory suffering for pedagogic purposes is ethically disputable. Yet, other potentially stressful experiences like simulated blindness or auditory hallucinations have been used [21–23]. We took extensive precautions to limit any untoward consequences of the dyspneic experience (participation strictly on a voluntary basis; detailed advance explanations on what to expect; the possibility to quit at any moment; the offer of psychological support on an as-needed basis). In the end, none of the participants reported regretting having participated in the course. Of note, the straw breathing challenge, one of the dyspnea induction techniques that we used, is often proposed to help the general public understand breathing difficulties, e.g., pediatric asthma) or to test anxiety sensitivity and reactivity to asthma-like sensations [32]. Although we did not collect explicit data about changes in empathy levels at one year, there are implicit indications that the experiential course may have positively influenced the participants’ propensity to empathetic solicitude towards dyspneic patients. Likewise, all of the participants considered that the experiential component of the course had been ‘useful to better apprehend what dyspneic patients experience’ (rated 7 [6–8] on a 0–10 numerical scale); they considered themselves ‘more attuned than before to what acute dyspnea represents for the patients’ (rated 8 [7–9]); they reported substantial increase in confidence and decrease in anxiety when managing dyspneic patients.

### Contribution of the personal dyspnea experience to course appraisal by the residents

Any of the course components (observation, personal experience, theory) could have contributed to our participants’ reactions and satisfaction with the course. Nevertheless, the personal experience of dyspnea seems to have played a particularly important role. Firstly, despite one or more previous theoretical courses on the topic, our participants mentioned incomplete satisfaction with their prior training on dyspnea. Secondly, we found statistically significant correlations between the overall course rating and several psychophysiological measures (intensity of breathing discomfort, dyspnea unpleasantness, MDP domains). This was not the case regarding the observation of the teacher’s dyspnea. Thirdly, the ‘experiential understanding’ variable (experimental dyspnea-related improvement in understanding dyspneic patients’ experience) was the only variable that remained significantly associated with the overall course rating in multivariate analysis. The apparent importance of ‘experiential understanding’ in changing the participants’ perspective is consistent with observations showing that breathing discomfort linked to the use of COVID-19 face masks relates to improved perception of the lived experience of patients with chronic respiratory disease [20].

### Consistency and discrepancies with the experimental dyspnea literature

Our data indicate that the experience of pain is more frequent than the experience of dyspnea in healthy young subjects, with pain remembered as more intense.Observing dyspnea in another person induced dyspnea in our participants, as reported previously [15], with a statistically significant correlation between vicarious dyspnea ratings and the ratings of self-perceived empathy [15]. Self-induced dyspnea was predominantly described as ‘air hunger’ in the sensory domain and associated with anxiety/frustration in the emotional domain. Ratings were particularly high, notably regarding dyspnea unpleasantness (median of 8). This observation is reminiscent of the high ratings associated, in the general public, with COVID-19 face mask-induced breathing discomfort, previously interpreted as illustrating the impossibility of imagining the intensity of a characteristic of which an individual has no direct experience [20]. We did not find differences according to the method used to self-induce dyspnea, in contrast with literature data differentiating inspiratory threshold loading and CO_2_ stimulation [33]. We do not have specific explanations for these observations.

### Strengths and limitations

The strengths of our study include its innovative design and the clear and reproducible results across two groups of residents from different specialties and separated temporally. The results demonstrate the feasibility of inducing experimental dyspnea in a large number of residents simultaneously using simple and transportable tools, making implementation in medical and nursing school programs realistic. However, the study also has several limitations. First, as in many studies of this type, changes in attitudes and beliefs could only be evaluated in a declarative manner, as well as empathy level, which leaves the possibility of bias. Second, there was a 50% attrition in the number of respondents at 1 year, so the long-term results should be interpreted with caution. Third, the study pertained to acute dyspnea and cannot readily be extrapolated to persistent dyspnea. Fourth, vicarious pain was not induced or assessed and could have been interesting to contextualize the residents’ reactions to observed dyspnea with respect to the corresponding reactions to pain. Finally, specific studies would be needed to assess the respective influences of the course components, and, importantly, to compare the experiential intervention to other types of intervention. We cannot rule out that other approaches like watching testimonials of dyspneic patients or expert-patients teaching could also be effective.

### Conclusions

In conclusion, the results of this pilot study support the pedagogical interest of dyspnea-targeted teaching programs incorporating an experiential element. This approach could help healthcare professionals better address the currently largely unmet patients’ dyspnea-related expectations [34].

## Supplementary Material

Supplemental Material

## Data Availability

The datasets analyzed during the current study are available from the corresponding author on reasonable request.
