# Supplementary material for: ‘Involve me and I learn’: an experiential teaching approach to improve dyspnea awareness in medical residents
Source: Med Educ Online. 2022 Oct 11;27(1):2133588. doi: 10.1080/10872981.2022.2133588 (PMC9559048; doi:10.1080/10872981.2022.2133588)
Supplement: Supplemental Material [file ZMEO_A_2133588_SM2344.docx]

**Electronic Supplement to**

**"*Involve me and I learn*": personal experience and dyspnea awareness in medical residents**

**ES1. Generic participants questionnaire**

**ES2. Psychophysiological reaction to the teacher's dyspnea**

**ES3. Multidimensional Dyspnea Profile ^35^**

**ES4. Immediate course evaluation questionnaire**

**ES5. One-year course evaluation questionnaire**

**ES6. Lexicometric analysis**

**ES7. Figure S1. First (panel A) and second (panel B) reasons advanced by the participants to not using morphine for the symptomatic relief of severe (NRS ≥ 8) pain or dyspnea**

**ES8. Figure S2. Distribution and intensity of the Multidimensional Dyspnea Profile sensory (upper panels) and emotional (lower panels) descriptors among the 53 participants who accepted to participate in the "experimental dyspnea" part of the study, immediately after being exposed to dyspnea.**

**ES9. Table S1. Comparison of the multidimensional dyspnea profile (MDP) scores between the three different dyspnea experimental devices**

**ES10. Figure S3. Distribution and intensity of the multidimensional dyspnea profile sensory (upper panels) and emotional (lower panels) descriptors among the 53 participants immediately after self-induced experimental dyspnea according to the type of dyspnea induction method.**

**ES1. Generic participants questionnaire (5 minutes)**

**General characteristics**

• How old are you?

• What is your gender?

**Personal history**

• During your life, have you ever felt pain (physical, not moral)? (Yes or No)

If Yes, on what occasion and what was the maximum intensity in your memory from 1: very low intensity to 10 extremely high intensity?

• During your life, have you ever felt dyspnea? (Yes or No)

If Yes, on what occasion and what was the maximum intensity in your memory from 1: very low intensity to 10 extremely high intensity?

• In general, regarding your personality, how would you rate your level of empathy from 0: no empathetic at all to 10: extremely empathetic?

**Academic formation**

• During your medical studies (second and third cycle of French medical studies) how many academic courses dedicated to dyspnea (e.g. pathophysiology, measurement, treatment) did you have to attend, before this experiential teaching course?

• In general, how would you rate, on a visual analog scale (VAS) from 0: not satisfied at all to 10: fully satisfied, the quality of the academic education (second and third cycle included) of dyspnea that you received during your medical studies (before this experiential teaching?)

• In general, would you like an improvement in the academic teaching of dyspnea during your medical studies (second and third cycle of French medical studies), VAS from 0: not motivated at all for an improvement to 10: fully motivated?

• At your current level of medical education (and before this experiential teaching), how would you rate your level of confidence in your ability to manage pain in a patient upon emergency room admission, VAS from 0: no confident at all, to 10: fully confident?

• At your current level of medical education (and before this experiential course), how would you rate your level of confidence in your ability to manage dyspnea in a patient upon emergency room admission, VAS from 0: no confident at all, to 10: fully confident?

**Attitudes and beliefs regarding pain and dyspnea**

• In your activity as a medical doctor, how do you quantify your propensity to look for pain in patients upon emergency room admission from 0: very low propensity to 10: very high propensity?

• In your activity as a medical doctor, how do you quantify your propensity to look for dyspnea in patients upon emergency room admission from 0: very low propensity to 10: very high propensity?

• In your activity as a medical doctor, how often do you prescribe morphine for severe pain relief?

- Never

- Very rarely (1/year)

- Sometimes (1/month)

- More than sometimes (>1/month)

• In your activity as a medical doctor, how often do you prescribe morphine for severe dyspnea relief?

- Never

- Very rarely (1/year)

- Sometimes (1/month)

- More than sometimes (>1/month)

• In your opinion, the right to pain relief is part of the French public health code? (Yes or No)

• In your opinion, the right to dyspnea relief is part of the French public health code? (Yes or No)

• Do you have an idea of the cut-off value that is considered clinically significant regarding pain (pain intensity that should require immediate relief) on a numerical rating scale from 0 to 10 (maximum intensity)?

If yes, what do you believe this cut-off is?

• Do you have an idea of the cut-off value that is considered clinically significant regarding dyspnea (dyspnea intensity that should require immediate relief) on a numerical rating scale from 0 to 10 (maximum intensity),

If yes, what is this cut-off?

• If your patient has pain rated 8/10 on the numerical rating scale:

*Are you initiating any specific treatments intended to relieve this pain? (Yes or No)

*What is your propensity to initiate an emergency morphine titration from 0: very low propensity to 10: very high propensity?

* Choose 2 factors that would contribute to not using morphine (score 1 and 2 from most to least important) among the following sentences:

1-Pain usually goes away by itself and does not need to be treated

2-Pain usually achieves adequate relief with other treatments

3-There is a lack of evidence for morphine efficacy in pain relief

4-Guidelines on morphine use for pain relief are inexistent or inappropriate for the emergency room setting

5- I have previous clinical experience that opioids for pain relief induced clinical deterioration

6-I only use morphine for pain relief in more advanced disease for end-of-life care settings

7-I am concerned by the risk of addiction/substance abuse

8-I am concerned by the potential negative side effects of morphine on breathing

9-I am concerned by the risk of confusion/delirium induced by morphine

10-I am afraid morphine will speed up the onset of death

• If your patient has dyspnea rated 8/10 on the numerical rating scale,

* Are you initiating any specific treatments intended to relieve this dyspnea? (Yes or No)

*What is your propensity to initiate an emergency morphine titration from 0: very low propensity to 10: very high propensity?

* Choose 2 factors that would contribute to not using morphine (score 1 and 2 from most to least important) among the following sentences:

1-Dyspnea usually goes away by itself and does not need to be treated

2-Dyspnea usually achieves adequate relief with other treatments

3-There is a lack of evidence for morphine efficacy in dyspnea relief

4-Guidelines on dyspnea use for dyspnea relief are inexistent or inappropriate for the emergency room setting

5- I have previous clinical experience that opioids in dyspnea relief induced clinical deterioration

6-I only use morphine for dyspnea relief in more advanced disease for end-of-life care settings

7-I am concerned by the risk of addiction/substance abuse

8-I am concerned by the potential negative side effects of morphine on breathing

9-I am concerned by the risk of confusion/delirium induced by morphine

10-I am afraid morphine will speed up the onset of death

**ES2. Psychophysiological reactions to the teacher's dyspnea**

• How much do you rate the teacher's dyspnea intensity on a numerical rating scale (NRS) from 0: very low to 10: extremely high?

• Have you experienced dyspnea yourself while watching the teacher? (Yes or No)

• If Yes, how do you quantify the dyspnea you felt on a NRS from 0: very low intensity to 10: extremely high intensity?

**ES3. The Multidimensional Dyspnea Profile (MDP) ^33^.**

The MDP consists of eleven items evaluating both the sensory and affective dimensions of dyspnea. It can generally be completed in 3 minutes or less. Before completing the questionnaires, participants are presented with a musical analogy to explain the difference between "sensory" and "affective", using the following script:

*On this page we ask you to tell us how unpleasant your breathing feels. On a later page, we will ask you about the intensity or strength of your breathing sensations. The distinction between these two aspects of breathing sensation might be made clearer if you think of listening to a sound, such as a radio. As the volume of the sound increases, I can ask you how loud it sounds or how unpleasant it is to hear it. For example, music that you hate can be unpleasant even when the volume is low, and will become more unpleasant as the volume increases; music that you like will not be unpleasant, even when the volume increases.*

One item (A1) assesses the unpleasantness of dyspnea on a 0-10 numerical scale anchored by "neutral" (0) and "unbearable" (10).

To assess the sensory dimension of dyspnea (SQ), participants are asked to report which of five items apply to their experience, which one of the five best apply to describe this experience, and to rate the five items on a 0-10 numerical rating scale from "none" to "as intense as I can imagine". These 5 items are listed below.

To assess the affective dimension of dyspnea (A2), participants are asked to rate five items qualifying the feelings associated to their perceived breathing difficulties, on a 0-10 numerical rating scale from "none" to "the most I can imagine". These 5 items are listed below.

| **Label** | **French sentences** | **English sentences** | **Coded variables** |
| --- | --- | --- | --- |
| A1 | Désagrément, caractère désagréable de vos sensations respiratoires | Unpleasantness or discomfort of your breathing sensations, how bad your breathing feels | **Intensity of the unpleasantness related to dyspnea** |
| SQ | Je dois fournir un travail ou un effort musculaire pour respirer  Je manque d’air ou j’étouffe ou je sens que j’ai besoin d’air  J’ai la sensation que ma poitrine et mes poumons sont serrés ou comprimés  Je dois me concentrer ou faire un effort mental pour respirer  Je respire fort | My breathing requires muscle work or effort  I am not getting enough air or I am suffocating, or I feel hunger for air  My chest and lungs feel tight or constricted  My breath requires mental effort or concentration  I am breathing a lot, rapidly, deeply, heavily | **Breathing-related sensations**  Muscle work    Air hunger    Chest tightness    Mental effort  Breathing a lot |
| A2 | Ma respiration me déprime  Ma respiration me rend anxieux(se)  Ma respiration me frustre  My respiration me rend en colère  My respiration m’effraie | My breathing makes me feel depressed  My breathing makes me feel anxious  My breathing makes me feel frustrated  My breathing makes me feel angry  My breathing makes me feel afraid | **Breathing-related emotions**  Depression  Anxiety  Frustration  Anger  Fear |

Several scores can be calculated from the above answers:

- sensory dimension: SQ obtained by summing the 5 sensory descriptors

- affective dimension: A1+A2

- immediate perception domain: SQ+A1

- emotional response domain: A2 obtained by summing the 5 emotional descriptors

**ES4. Immediate course evaluation questionnaire**

• To what extent did your personal experience of dyspnea change your understanding of this symptom?

- Not at all

- A little

- A lot

- Totally

• To what extent did your personal experience of dyspnea make you better understand what dyspneic patients feel?

NRS from 0: absolutely not to 10: considerably?

• Finally, please give a grade from 0 to 20 for the course!

**ES5. One-year course evaluation questionnaire**

• Do you think that the type of experiential teaching to which you were exposed in November 2019, is a good method to become familiar with dyspnea and thus be able to better understand what acute dyspnea represents for the patient? (Yes or No)

• Since the experiential teaching of dyspnea that you received in November 2019, do you consider yourself (VAS from 0: not at all, to 10: totally) more aware of the patients’ suffering that represents dyspnea and more prone to looking for dyspnea in these patients?

• Since the experiential teaching of dyspnea that you received in November 2019, to what extent, VAS from 0: not at all to 10: very much, do you feel more confident in the management of acute dyspnea in the emergency room?

• Since the experiential teaching of dyspnea that you received in November 2019, to what extent, VAS from 0: not changed, 10 greatly reduced, do you have the impression that the management of acute dyspnea in an emergency room patient causes you less anxiety than before?

• One year after this experiential teaching of dyspnea, give us the 3 words that you think best describe the impact it has had on you and your practice.

**ES6. Lexicometric analysis (Methods, Results, Figure)**

**Methods**

Words or expressions chosen by each participant after their self-induced dyspnea experience were lemmatized and merged as individual verbatims to perform a correspondence factorial analysis followed by descending hierarchical classification. The creation of a paired binary variable (e.g. men and high empathy) was necessary to enter these binary variables in correspondence factorial analysis. These paired binary variables had therefore four possible issues depending on the presence or absence of each component.

**Results**

The lexicometric analysis of the participants’ verbatim identified three semantic classes (Figure). Class 1 had a "unpleasant" connotation" ("unpleasant", "adaptation", "limitation"), class 2 had a more "emotional” connotation ("anxiety", "frustration"), class 3 was more neutral (“die”, “air”, “miss”) and class 4 was more “sensory” connotation ("keep calm", "discomfort"). There was no statistically significant association between these classes and other variables. Nevertheless, words from class 2 and 4 were more frequent in high empathy group, while words from class 3 were more frequent in the low empathy group. Class 3 and class 4 were diametrally opposed regarding the estimation of the teacher’s dyspnea intensity (low intensity for class 3 and high intensity for class 4)

**Figure. Illustration of the results of the lexicometric analysis performed on the verbatim collected immediately after exposure to self-induced dyspnea. Four semantic classes were identified.**

**ES7. Figure S1. First (panel A) and second (panel B) reasons advanced by the participants for not using morphine for the symptomatic relief of severe (NRS ≥ 8) pain or dyspnea**

* p<0.05

NRS, numerical rating scale (from 0: minimal to 10: maximal intensity)

**ES8. Figure S2. Distribution and intensity of the Multidimensional Dyspnea Profile sensory (upper panels) and emotional (lower panels) descriptors among the 53 participants who accepted to participate in the "experimental dyspnea" part of the study, immediately after being exposed to dyspnea.**

Grey bars indicate the proportion (%) of subjects that chose the sensation or emotion. White boxes represent the median and the interquartile interval, whiskers the 95% confidence interval.

**ES9. Table S1. Comparison of the multidimensional dyspnea profile (MDP) scores between the three different dyspnea experimental devices**

| **MDP scores** | **All**  **n = 53** | **Inspiratory resistance**  **n = 31** | **CO_2_**  **rebreathing**  **n = 16** | **Inspiratory threshold loading**  **n= 6** | ***P***  **(KW)** |
| --- | --- | --- | --- | --- | --- |
| **A1 (unpleasantness intensity), *NRS*** | 8 [6–9] | 8 [6–9] | 8 [6–10] | 9 [6–9] | 0.559 |
| **A2 (emotional descriptors NRS sum)** | 16 [8–22] | 13 [7–22] | 18 [8–24] | 22 [17–28] | 0.189 |
| **Affective dimension (A1+A2)** | 23 [14–31] | 21 [13–31] | 26 [13–31] | 30 [27–37] | 0.225 |
| **SQ (sensory descriptors NRS sum)** | 33 [25–29] | 30 [24–38] | 35 [24–40] | 37 [29–44] | 0.311 |
| **Immediate perception domain (SQ+A1)** | 41 [32–46] | 39 [30–46] | 43 [29–48] | 44 [36–54] | 0.316 |

Continuous variables are expressed as median (interquartile range)

NRS, numerical rating scale (from 0 minimal value to 10 maximal value); KW, Kruskal-Wallis test.

**ES10. Figure S3. Distribution and intensity of the multidimensional dyspnea profile sensory (upper panels) and emotional (lower panels) descriptors among the 53 participants immediately after self-induced experimental dyspnea according to the type of dyspnea induction method.**

* p<0.05 with Kruskal-Wallis test.

Tube, inspiratory resistance through plastic tube; CO2: CO_2_ rebreathing with a 2L single use bag; ITL, inspiratory threshold loading
